# Traditional Chinese medicine for HIV-related chronic comorbidities: evidence and potential therapeutic mechanisms

**DOI:** 10.3389/fphar.2025.1698434

**Published:** 2026-01-08

**Authors:** Jiahe Li, Liran Xu, Xue Ding, Xiuxia Ma, Pengyu Qian, Nao Qiu, Jingyu Yue

**Affiliations:** 1 Department of The First Clinical Medical College, Henan University of Chinese Medicine, Zhengzhou, China; 2 Department of AIDS Clinical Research Center, The First Affiliated Hospital of Henan University of Chinese Medicine, Zhengzhou, China; 3 Department of Medical, The First Affiliated Hospital of Henan University of Chinese Medicine, Zhengzhou, China

**Keywords:** HIV infections, comorbidity, Chinese botanical drugs, complementary, alternative

## Abstract

HIV-related chronic comorbidities negatively impact health and pose a global public health challenge, necessitating the development of new drugs and therapeutic approaches. Traditional Chinese medicine (TCM) formulations, used for over 2,000 years, are increasingly being studied for the management of these conditions. Recently, an increasing number of studies have investigated the clinical management of HIV-related chronic comorbidities. In this review, we discuss the clinical applications and therapeutic mechanisms of Chinese botanical drugs in treating HIV-related chronic comorbidities, including cardiovascular diseases, chronic kidney disease, neurocognitive disorders, metabolic syndrome, and osteoporosis. We illustrate that various key metabolites, such as tanshinones, berberine, and astragalus polysaccharide, show beneficial effects across these systems, primarily by modulating key pathogenic drivers. The therapeutic mechanisms elucidated primarily involve inhibition of viral replication, modulation of inflammatory pathways (e.g., NF-κB, MAPK, and TLR4), restoration of intestinal mucosal integrity, and rebalancing of immune homeostasis (e.g., Th17/Treg balance). This preclinical evidence supports the effectiveness of these Chinese botanical drugs as complementary and alternative therapeutic options; however, most of the available evidence is based on *in vitro* and animal studies, and their clinical translational value is significantly limited. Further rigorous clinical trials are needed to verify the efficacy and safety of Chinese botanical drugs in treating people living with HIV.

## 1 Introduction

Efficacious antiretroviral therapy (ART) and successful global treatment initiatives have significantly increased the life expectancy of people living with HIV (PLWH) and receiving ART. Consequently, HIV-related illnesses are no longer the primary health threat for these individuals (The Lancet Healthy L, 2022; Rasmussen et al., 2015). In the ART era, morbidity and mortality in PLWH are now predominantly driven by several chronic comorbidities, including cardiovascular diseases (CVDs) and cancer (Deeks et al., 2013). ART has improved the life expectancy of PLWH, which is currently only 3 years shorter than that of adults without HIV infection. However, the age of onset of comorbidities is 16 years earlier in PLWH than in adults without HIV infection. Common comorbidities, which are diagnosed as early as age 34 and do not improve with early ART (Marcus et al., 2020), affect the lifespan and quality of life of PLWH. Half (50%) of the long-term survivors of HIV develop two or more chronic diseases, typically including CVDs, metabolic syndrome (MetS), and neurocognitive disorders. There are growing concerns that HIV-related chronic comorbidities may eventually overwhelm some healthcare systems. These comorbidities are increasingly common among PLWH, with the prevalence of multimorbidity increasing from 8.2% to 22.4% between 2000 and 2009 (Wong et al., 2018). Furthermore, as AIDS-related mortality declines, the rates of non-AIDS-related deaths are rising (Smith et al., 2014), underscoring the importance of preventing chronic comorbidities in PLWH.

Traditional Chinese medicine (TCM) formulations have been used to treat diseases in China for more than 2,000 years. TCM remains an important part of the Chinese healthcare system, with medical practitioners and patients commonly using TCM to prevent and treat various diseases. Research has shown that TCM formulations exhibit significant biological activities, including anti-inflammatory, antiviral, antitumor, antioxidant, hypolipidemic, and immunomodulatory effects (Wang et al., 2024; Feng et al., 2020; Zeng et al., 2019). The efficacy and safety of TCM in the management of AIDS have been demonstrated and resulted in therapeutic outcomes, including reductions in HIV-related symptoms and adverse side effects of ART, along with improvements in the quality of life (Liu et al., 2015). However, the pharmacological mechanisms by which botanical drugs treat HIV and HIV-related chronic comorbidities have not been elucidated. In this review, we discuss the relationship between botanical drugs used in TCM and HIV-related chronic comorbidities, with a focus on their therapeutic effects and underlying mechanisms of action. By synthesizing current evidence, we aim to offer new insights into the treatment of HIV-related chronic comorbidities.

## 2 Results

### 2.1 TCM and HIV-related chronic comorbidities

Metabolites, such as polyphenols, terpenoids, saponins, and alkaloids, have been isolated from TCM and shown to have beneficial effects on the comorbidities. A search was conducted on China National Knowledge Infrastructure, Wanfang, Chinese Biomedical Literature Database, PubMed, Embase, and Medline for studies on the effects of botanical drugs on HIV-related chronic comorbidities published up to November 2025. The data are presented in Table 1.

**TABLE 1:** Representative examples of the effects of TCM metabolites on comorbidities and the potential mechanisms.

Comorbidity	Key metabolite	Representative botanical drug	Complete representative botanical drug	Family	Beneficial effect	Potential mechanism	Experimental models used	References
Cardiovascular diseases	*Tanshinones*	*Salvia miltiorrhiza*	*Salvia miltiorrhiza Bunge*	*Lamiaceae*	Anti-inflammatory, antioxidant, anti-atherogenic; cardioprotective, antithrombotic, vasodilator, and proangiogenic	Inhibiting NF-κB, Nox, and JAK/STAT pathways, locking Kþ channels, increasing the synthesis of NO and epoxyeicosatrienoic acids, inhibiting calcium ions, and anti-apoptosis	In rats (hypercholesterolemic): 35, 70 mg/kg/day; high-cholesterol model group In rabbits (high-fat diet fed): 3, 10, and 30 mg/kg/day; high-fat diet model group In mice (ApoE−/−): 10, 30, and 90 mg/kg/day; high-fat diet model group In rabbits (high-fat diet fed): 6.25, 15, and 37.5 mg/kg/day; high-fat diet model group In rat (carotid artery balloon injury): 13.3, 40, and 120 mg/kg/day; balloon injury model group In vitro (rat mesenteric arteries): 1 μM−100 μM; vehicle (0.2% DMSO)/1 μM prazosin/100 μM NG-nitro-L-arginine (L-NNA)/1 mM L-arginine	Gao et al. (2012)
​	*Baicalin*	*Scutellaria baicalensis*	*Scutellaria baicalensis Georgi*	*Lamiaceae*	Antioxidant, anti-inflammatory, improving endothelial function; and cardioprotective	Free-radical scavenging, inhibiting xanthine oxidase; inhibiting lipoxygenase, inhibiting endoplasmic reticulum stress-induced apoptosis, and inhibiting the calcineurin/NFATc3 pathway	In cell (neonatal rat cardiomyocytes): baicalin: 0 mM–50 mM pretreatment)/tunicamycin: 100 ng/mL (treatment); control group/tunicamycin group] In isolated rat (mesenteric arteries): baicalin (1 μM–100 μM)/baicalein (1 μM–50 μM) *In* *vitro* (rat mesenteric arteries endothelium-denuded rings): 10μM; control (endothelium-denuded rings)	Huang et al. (2005), Shen et al. (2014)
​	*Berberine*	*Rhizoma Coptidis*	*Coptis chinensis Franch*	*Ranunculaceae*	Positive inotropic activity, antioxidant, and anti-apoptotic	Increasing phosphorylation of the pro-apoptotic factor, reducing pro-inflammatory mediators, locking Kþ channels, activating AMP-activated protein kinase and PI3K/Akt pathways and upregulation of miR-340-5p	In dogs (ischemic left ventricular failure): 1 mg/kg i.v. + 0.2 mg/kg/min infusion In rats (pressure overload): 10 mg/kg/d, p.o.; vehicle control In rats (2K2C renovascular hypertension): 5 and 10 mg/kg/day, p.o.; vehicle control In rats (isoproterenol-induced heart failure): 20 mg/kg/day (BBR) + 20 mg/kg/day (ginseng saponins); captopril group In cell (H9C2 rat cardiomyocytes): 50 μM; normal cultured cells/H/R model group In rats (Sprague–Dawley): 200 mg/kg/day; 0.5% CMC-Na solution/MIRI model group) In humans: 300 mg, t.i.d, 3 months; patients with stable coronary artery disease not taking BBR	Feng et al. (2019), Long et al. (2023), Han et al. (2022)
​	*Ginsenosides*	*Radix Ginseng*	*Panax ginseng C. A. Mey*	*Araliaceae*	Anti-inflammatory, antioxidant, cardioprotective, and proangiogenic	Inhibiting NF-κB and stabilizing hypoxia-inducible factor-1-activating PI3K/Akt pathway	In rats (Sprague–Dawley CHF model): 100 mg/kg/day, i.v., 8 weeks; sham operation group (NS)/CHF model group (NS)/captopril group *In vitro* (rat CMECs, inflammation model): 0.5 mg/mL YQFM or 100 μmol/L metabolites; TNF-α stimulation group/dexamethasone (10 μmol/L)	Xing et al. (2013)
Chronic kidney disease	*Astragalus polysaccharide*	*Astragalus membranaceus*	*Astragalus membranaceus (Fisch.) Bunge*	*Fabaceae*	Anti-inflammatory, antioxidative, immunomodulatory, and kidney protection	Inhibiting TLR4/NF-κB/MAPK pathway, affecting the TGF-β/Smad pathway, and anti-apoptosis	In mice (C57BL/6): (1, 3, and 5 mg/kg/day for 3 consecutive days; saline/LPS model group In cell (HK-2 cells): 100 μg/mL; PBS/LPS model group In rats (SD rats): 200, 400, and 800 mg/kg/day, 4 weeks; vehicle/STZ model group) In cell (MPC5 podocytes): 5% APS-medicated serum, 48 h; HG model group/TAK-242 group	Guo et al. (2023), Sun et al. (2021)
​	*Triptolide*	*Tripterygium wilfordii*	*Tripterygium wilfordii Hook. F*	*Celastraceae*	Anti-inflammatory, immunosuppressive, and podocyte-protective	Activating kindlin-2 and EMT-related TGF-β/Smad signaling pathway	In cell (rat mesangial cells): 10 μg/L, co-treatment with TGF-β1 (10 μg/L); normal control/TGF-β1 (10 μg/L) model group In cell (rat mesangial cells): 0.4, 2, and 10 μg/L; normal control/TGF-β1 (10 μg/L) model group In rats (SD rats): 0.2 mg/kg/day; normal control/CSS model group In mice (db/db mice): 50 and 75 μg/kg/day, 12 weeks; negative control/diabetic control	Cao et al. (2015), Ren et al. (2022)
Neurocognitive disorders	*Tetramethylpyrazine*	*Ligusticum chuanxiong*	*Ligusticum chuanxiong Hort*	*Apiaceae*	Neuroprotective	Inhibiting apoptosis	In rats (Wistar): 20 mg/kg, i.p., q.d.; vehicle (cremophor:ethanol:saline = 1:1:4) In rat (brain homogenates): 0.5 mmol/L–5 mmol/L; 0.5% DMSO	Chang et al. (2007)
​	*Glycyrrhizin*	*Glycyrrhizae*	*Glycyrrhiza uralensis Fisch. ex DC.*	*Fabaceae*	Anti-inflammatory, antioxidant, and apoptosis	Downregulating HMGB1/TLR4/NF-κB p65 signaling and inhibiting HMGB1/TLR4/NF-κB	In rats (Wistar): 50 mg/kg, i.p., q.d., 3 weeks; saline	Gendy et al. (2023)
Metabolic syndrome	*Ligupurpuroside A and acteoside*	*Ligustrum robustum*	*Ligustrum robustum (Roxb.) Blume*	*Oleaceae*	Anti-inflammatory and improving metabolism	reshaped the gut microbiota structure	In mice (C57BL/6J): 200 mg/kg/d, p.o., 16 weeks; vehicle/HFD group	Chen et al. (2021)
​	*Breviscapine*	*Erigeron breviscapus*	*Erigeron breviscapus (Vant.) Hand.-Mazz*	*Asteraceae*	Anti-inflammation and anti-fibrosis	Inhibiting TAK1 signaling	In mice (C57BL/6J): 15 and 30 mg/kg, q.d., p.o., 8 weeks; vehicle/HFD group In mice (C57BL/6J): 15 and 30 mg/kg, q.d., p.o., 8 weeks; vehicle/HFHC group In mice (C57BL/6J): 30 mg/kg, q.d., p.o., 4 weeks; vehicle/MCD group *In vitro* (primary hepatocytes): 50 and 100 μM; vehicle/PO group *In vitro* (L02 hepatocytes): 50 and 100 μM; vehicle/PO group	Lan et al. (2022)
​	*Danthron*	*Rhubarb*	*Rheum palmatum L*	*Polygonaceae*	Anti-inflammation, antibacterial, and detoxification	Activating the interplay between PPARα/RXRα heterodimer and adiponectin receptor 2	In mice [(C57BL/6J) fed with high-fat diet (HFD)]: 10 mg/kg/day bodyweight (mpk) (HFD duration 16 weeks, DAN starting at week 10 for 6 weeks); vehicle and normal chow (NC) *In vitro* (3T3-L1-derived adipocytes and HepG2 cells): 10 and 20 μM; Si-Ctrl (scrambled siRNA), vehicle/control (DAN0)	Ma et al. (2021)
Osteoporosis	*Corynoline*	*Corydalis bungeana*	*Corydalis bungeana Turcz.*	*Papaveraceae*	Restoring bone mass, improving microarchitecture, and reducing the ROS levels	Inhibiting NF-κB/MAPK signaling and enhancing the protein stability of the Nrf2	*In vitro* (bone marrow-derived macrophages (BMMs)): 2, 4, and 8 μM; vehicle/RANKL group In mice (C57BL/6 (OVX model)): 10 and 20 mg/kg, i.p. every 2 days, 8 weeks; vehicle/OVX group In mice [C57BL/6 (OVX model)]: 20 mg/kg Cor +30 mg/kg ML385, i.p. every 2 days, 8 weeks; vehicle/OVX group	Jin et al. (2024)
​	*Icaritin*	*Epimedium*	*Epimedium L*	*Berberidaceae*	Inhibiting osteoclastogenesis, attenuating the bone loss	Downregulating transcription factors activated T-cell cytoplasm 1 (NFATc1) and c-fos	*In* *vitro* [bone marrow macrophages (BMMs)]: 0.01, 0.1, and 1 μM; DMSO (vehicle) *In vivo* (C57BL/6 female mice, OVX model): 10 mg/kg, i.p., every 2 days, 6 weeks; vehicle	Huang et al. (2023)
​	*Ginsenoside Rb2*	*Panax ginseng*	*Panax ginseng C. A. Mey*	*Araliaceae*	Inhibiting osteoclastogenesis, attenuating the bone loss	Inhibiting osteoclastogenesis and inhibiting NF-κB/MAPK/STAT3 pathways	*In* *vitro* (RAW 264.7 cells): 0.1, 1, and 10 μM; vehicle (DMSO) *In* *vitro* (RAW264.7 cells): 0.1 and 1 μM; RANKL (50 ng mL^−1^)] *In* *vitro* (bone marrow macrophages (BMMs)): 0.1 and 1 μM; RANKL (50 ng mL^−1^) In mice (C57BL/6J male, ORX model): 5 and 10 mg kg^−1^, i.p., every 2 days, 8 weeks; PBS (vehicle)	Ma et al. (2024), Cong et al. (2017)
​	*Catalpol*	*Rehmannia*	*Rehmannia glutinosa (Gaertn.) DC.*	*Orobanchaceae*	Promoting osteoclast apoptosis	Upregulating the expression of Sirt6, ERα, FasL, cleaved-caspase 8, cleaved-caspase 3, and Bax and downregulating the expression of NFATc1, Ctsk, and Oscar	In rats (female OVX): 5, 10, and 20 mg/kg/day, p.o., 12 weeks; distilled water (vehicle)/alendronate (2.5 mg/kg)	Chen et al. (2024)

### 2.2 TCM and HIV-related CVDs

PLWH are twice as likely to develop CVDs as people without HIV infection, leading to a tripling of the global burden of HIV-related CVDs over the past 20 years (Shah et al., 2018). Among these chronic comorbidities, the prevalence of CVDs increased by approximately fourfold between 2004 and 2014, with the proportion of PLWH at high or extremely high risk of CVDs more than doubling, according to the Data Collection on Adverse Events of Anti-HIV Drugs coronary risk score (Bonnet et al., 2020). Dyslipidemia, smoking, hypertension, diabetes, and obesity are common factors that contribute to the increase in the risk of CVDs (Guo et al., 2017). HIV infection impacts the heart and arterial system through underlying mechanisms including the continued expression of HIV-encoded proteins on immune and vascular cells, immunodeficiency, gut microbial translocation, chronic inflammation, and immune cell activation (Hsue and Waters, 2019).

Current evidence indicates the effectiveness of some TCM formulations as complementary and alternative therapeutic options to prevent CVDs. Specifically, TCM has shown effectiveness in alleviating myocardial perfusion abnormalities and neurological deficits, along with improving cardiac remodeling and function, demonstrating good cardiovascular safety profiles (Hao et al., 2017). Baicalin from *Scutellaria baicalensis*, an effective antioxidant, exerts antithrombotic and anti-inflammatory effects on endothelial cells and has been shown to protect myocardial cells by interfering with endoplasmic reticulum stress-induced apoptosis (Huang et al., 2005; Shen et al., 2014). Similarly, tanshinones—lipophilic diterpenoid metabolites primarily isolated from the roots of *Salvia miltiorrhiza*—exert cardioprotective effects on cardiac myocytes (Gao et al., 2012), at least in part by scavenging oxygen free radicals, inhibiting the calcineurin/NFATc3 pathway, and upregulating miR-223-5p and increasing the Bcl-2/Bax ratio (Li et al., 2023; Tan et al., 2011; Fu et al., 2007). Ginsenosides in TCM, a class of saponins found in ginseng, are used to treat heart failure. Evidence from experimental studies and clinical trials has shown that saponins exert anti-inflammatory, antioxidant, and proangiogenic effects (Meng et al., 2022; Yin et al., 2011). Saponins may prevent cardiomyocyte apoptosis in a protein kinase A-dependent manner (Wang et al., 2013). Berberine from Rhizoma Coptidis provides cardiovascular benefits through its positive inotropic activity, increased phosphorylation of the pro-apoptotic factor Bad, reduced production of pro-inflammatory mediators [interleukin (IL)-6, IL-1β, and tumor necrosis factor (TNF)-α], attenuation of oxidative stress, lowering of blood pressure, and anti-apoptotic effects (Dai et al., 2022; Feng et al., 2019). Protective effects of berberine in CVDs are also exerted through global modulation of long non-coding RNA (lncRNA) and mRNA expression and upregulation of miR-340-5p (Long et al., 2023; Han et al., 2022). Finally, berberine has been shown to alleviate endothelial junction dysfunction by inhibiting inflammasome activation. Collectively, these findings support a crucial link between TCM and CVD prevention and treatment.

### 2.3 HIV-related chronic kidney disease (CKD)

Kidney injury is an important complication of HIV infection. CKD has since emerged as a serious health concern and burden among PLWH, driven by both HIV-specific and traditional risk factors (Achhra et al., 2016; Stanifer et al., 2014). Some research studies suggest that the incidence of CKD among PLWH is almost four times that in HIV-negative populations (Rasmussen et al., 2015). Hypertension, dyslipidemia, CVDs, diabetes, and recent low CD4^+^ cell count have all been associated with the risk of CKD in PLWH (Gao et al., 2023). CKD is a major risk factor for end-stage renal disease and all-cause mortality, and a deeper understanding of its pathogenesis in PLWH is essential for improving long-term outcomes.

TCM is an effective alternative treatment option for CKD. Astragalus polysaccharide, one of the polysaccharide bioactive metabolites of *Astragalus membranaceus*, exhibits anti-inflammatory, antioxidative, and immunomodulatory properties against kidney injury through mechanisms related to relieving inflammatory responses and inhibiting M1 macrophage polarization. These effects are mediated through modulation of the Toll-like receptor 4 (TLR4)/NF-κB pathway, the lncRNA Gm41268/PRLR, and the cGAS/STING signaling pathway (Guo et al., 2023; Chen Z. et al., 2023; Sun et al., 2024). Triptolide extracted from *Tripterygium wilfordii* has been extensively used in China for treating CKD. Triptolides have been shown to exert strong anti-inflammatory, antioxidative, immunosuppressive, and immunomodulatory effects in many diseases (Yang J. et al., 2022; Qiu and Kao, 2003). Recent studies suggest that triptolides inhibit mesangial cell proliferation in immunoglobulin (Ig) A nephropathy via the CARD9/p38 MAPK pathway (Zhao et al., 2022). Triptolides reportedly exert a strong anti-proteinuric effect by reducing podocyte permeability through TET2-mediated hydroxymethylation of the tight junction protein ZO-1 (Tang et al., 2024). Saikosaponin A extracted from *Bupleurum falcatum* has been reported to possess anti-inflammatory and antioxidative activities in CKD. Saikosaponins have also been shown to attenuate fibrosis in kidney disease by regulating the Hedgehog and transforming growth factor (TGF)-β1/BMP7/Gremlin1/Smad pathways (Ren et al., 2020; Ruiqi et al., 2021). Taken together, these studies highlight TCM as an important entry point for the treatment of HIV-related CKD.

### 2.4 HIV-associated neurocognitive disorders (HANDs)

In the era of ART, the most severe HIV-associated neurocognitive disorders (HANDs) are rare, but minor forms of impairment remain common (Heaton et al., 2010). HIV can enter the central nervous system during the early stage of infection. Persistent HIV infection and inflammation in the central nervous system may contribute to the development of HANDs (Saylor et al., 2016). Subsequently, the brain becomes a repository for continuous HIV replication, thereby limiting the opportunities for treatment or eradication (Fois and Brew, 2015). The development of HANDs in PLWH receiving ART negatively impacts their survival and quality of life, along with daily functions (Hea et al., 1994).

Elucidation of the pharmacological properties and mechanisms of TCM in the treatment of neurocognitive disorders holds promise for therapeutic development (Chen et al., 2018). Tetramethylpyrazine isolated from *Ligusticum chuanxiong* has been shown to ameliorate cerebral ischemia–reperfusion injury through reduction of neuroinflammation in mice (Xiao et al., 2010; Chang et al., 2007). Another study showed that tetramethylpyrazine attenuates neurocognitive dysfunction by inhibiting the inflammatory response and increasing water molecule diffusivity and cerebral blood perfusion in the rat brain (Guangming et al., 2019). Glycyrrhizin, a saponin triterpenoid derived from the dried roots of Glycyrrhizae, combined with a sub-anesthetic dose of esketamine, was shown to inhibit HMGB1, TLR4, and NF-κB, improve learning and memory ability, and reduce hippocampal neuroinflammation in mice (Bin et al., 2024). Further exploration of the mechanisms of action of such herbal medicines could be of great significance, guiding future research on neurocognitive disorders.

### 2.5 HIV-associated metabolic syndrome (MetS)

Modern lifestyle changes have significantly increased the prevalence of MetS, which poses a major health hazard (Yang C. et al., 2022). HIV-encoded proteins and some ART agents induce dysfunction in adipocyte health, leading to dyslipidemia and insulin resistance, thereby contributing to the development of MetS in PLWH (Masenga et al., 2020). The rising prevalence of MetS among PLWH has gained global attention (Trachunthong et al., 2024), underscoring the urgent need to delineate therapeutic strategies. Recent research highlights the potential of TCM metabolites in the treatment of MetS. *Ligustrum robustum* extract (ligupurpuroside A and acteoside) has shown potential for managing MetS by inhibiting inflammatory responses, reducing insulin resistance, and improving metabolism in mice (Chen et al., 2021). Breviscapine, a natural flavonoid prescription drug isolated from *Erigeron breviscapus*, exerts metabolic effects through direct inhibition of TGF-β-activated kinase 1 signaling (Lan et al., 2022). Danthron, an anthraquinone derivative extracted from rhubarb, exhibits preventive effects against MetS by activating the interplay between PPARα/RXRα heterodimer and adiponectin receptor 2 (Ma et al., 2021).

HIV-associated diabetes requires a differentiated approach; type 2 diabetes mellitus (T2DM) is driven by metabolic toxicity (Oliveira et al., 2022). Dioscoreae Rhizoma exerts its effects primarily through its active metabolite *Dioscorea polysaccharide* (DPS); DPS ameliorates insulin resistance by increasing glucose absorption and GLUT2 expression while activating insulin receptor substrate phosphorylation and elevating p-Akt levels. In animal studies, DPS demonstrates hypoglycemic properties and protects pancreatic β cells against oxidative damage by enhancing antioxidant enzyme activity (Fan et al., 2015; Ding et al., 2013). Mechanistically, it modulates the PI3K/Akt signaling pathway by regulating proteins such as FoxO. Ginseng Radix et Rhizoma, through its active component ginsenoside Rb_1_ (Zhou et al., 2023; Xiong et al., 2010), treats T1DM by regulating glucose and lipid metabolism through the suppression of adipogenic genes such as *PPARγ* (Shin and Yoon, 2018). It protects pancreatic β cells, restores insulin secretion, and modulates immune homeostasis (Hong et al., 2012). Ginsenosides also reverse the symptoms of gut microbiota dysbiosis and reduce food intake via hypothalamic Akt/PI3K signaling (Zhang et al., 2019). Furthermore, ginseng mitigates oxidative stress and endothelial injury through the Nrf2 pathway (Wang et al., 2022), improves diabetic cardiomyopathy by regulating calcium signaling, and enhances renal function while reducing inflammation (Qin et al., 2019; Chen XM. et al., 2023). Berberine (Rhizoma Coptidis) has emerged as a critical candidate for HIV-associated T2DM, activating the AMPK pathway to improve glucose uptake similarly to metformin, and formulas containing *Astragalus* help repair the intestinal mucosal barrier to reduce metabolic endotoxemia (Ma et al., 2016). Research indicates that protease inhibitors (PIs) inhibit the activity of the GLUT4 transporter, leading to hyperglycemia (Koster et al., 2003). Experimental data show that berberine activates the AMPK (adenosine monophosphate-activated protein kinase) pathway, thus significantly improving insulin resistance induced by ritonavir in macrophages and adipocytes (Huang et al., 2006; Lee et al., 2006). HIV infection causes chronic low-grade inflammation. Berberine has been shown to inhibit the IKKβ/NF-κB signaling pathway, thereby reducing inflammation-induced insulin signal blockade (Li, 2018). Such evidence highlights the therapeutic potential—and the need for safety-conscious application—of Chinese herbal remedies in managing HIV-associated MetS.

### 2.6 HIV-associated tuberculosis (TB)

The clinical management of HIV–TB co-infection is frequently complicated by cumulative drug hepatotoxicity, drug–drug interactions, and the risk of immune reconstitution inflammatory syndrome (IRIS). Active metabolites derived from TCM offer a promising complementary strategy. Certain metabolites exhibit dual-target activities against both pathogens. Curcumin has been shown to suppress HIV-1 replication by inhibiting the Tat transactivator and NF-κB pathway while concurrently enhancing the intracellular elimination of *Mycobacterium tuberculosis* through the induction of macrophage autophagy and apoptosis (Prasad and Tyagi, 2015; Bai et al., 2016). Its potent anti-inflammatory properties may also help attenuate the cytokine storm associated with IRIS. Baicalin demonstrates the ability to block HIV-1 entry and promote a Th1-dominant immune response, which is essential for effective TB containment (Cai et al., 2008; Kitamura et al., 1998). The combined regimen of ART and anti-tuberculosis drugs often leads to severe liver injury. Glycyrrhizic acid has been proven to mitigate oxidative stress and reduce drug-induced liver injury caused by agents such as rifampicin and isoniazid (Dong et al., 2014). Through hepatoprotective effects, TCM can improve patient tolerance and adherence to standard regimens. Therefore, integrating these bioactive metabolites as adjuvants represents a valuable avenue for optimizing HIV–TB co-infection outcomes.

### 2.7 HIV-associated cancers

Emerging evidence elucidates that specific active metabolites derived from TCM exert potent therapeutic effects on HIV-associated cancers by targeting the intricate cross talk between viral proteins and oncogenic signaling pathways. These phytochemicals often operate through a multi-target mechanism that addresses both the viral persistence and the tumorigenic microenvironment (Wang et al., 2021). TCM metabolites directly inhibit the oncogenic potential of HIV viral proteins, particularly Tat and Nef, which are known to promote angiogenesis and cellular proliferation (Isaguliants et al., 2021). Curcumin has been shown to suppress the HIV-1 Tat-induced transactivation of viral replication and block the Tat-mediated proteasomal degradation of tumor-suppressor proteins such as p53. This inhibition effectively disrupts the Tat-driven transformation of B cells in non-Hodgkin lymphoma (Ali and Banerjea, 2016). Similarly, epigallocatechin-3-gallate, the most abundant polyphenol in green tea, demonstrates dual efficacy by inhibiting the latent membrane protein 1 in EBV-co-infected lymphomas and suppressing the ROS/MAPK/NF-κB signaling axis, which is chronically activated in the HIV-infected host (Hauber et al., 2009). TCM metabolites play a critical role in remodeling the immunosuppressive tumor microenvironment, which is particularly relevant in HIV patients. Resveratrol has been observed to induce apoptosis in Kaposi sarcoma cells by downregulating the expression of viral FLICE-inhibitory protein (vFLIP) and inhibiting the PI3K/Akt/mTOR pathway, which is frequently hyperactivated in AIDS-defining malignancies (Singh and Pai, 2014). Furthermore, baicalin and triptolide exert anti-inflammatory effects by inhibiting the nuclear translocation of NF-κB and reducing the secretion of pro-inflammatory cytokines (IL-6 and TNF-α) that fuel the growth of non-AIDS-defining cancers (Janssens et al., 2024). Collectively, these findings suggest that TCM metabolites do not merely serve as adjunctive support but also function as sophisticated molecular interceptors that sever the link between HIV-induced immunodeficiency and neoplastic progression.

### 2.8 HIV-associated osteoporosis

Osteoporosis, through its association with fragility fracture, has emerged as an important comorbidity of HIV infection. The prevalence of osteoporosis in PLWH is more than three times higher than that in HIV-negative individuals (Brown and Qaqish, 2006). ART, protease inhibitor exposure, HIV/hepatitis C virus (HCV) co-infection, immune activation, and inflammation are major contributors to osteoporosis in PLWH (McGinty et al., 2016; Bedimo et al., 2016; Ward et al., 2023). T-cell repopulation and immune reconstitution constitute putative mechanisms of ART-induced bone loss (Ofotokun et al., 2015). Bone homeostasis is partially regulated by immune system cells through complex interactions with the RANK/RANKL/OPG axis. However, disturbances of the normal functioning of B cells, T cells, and monocytes in HIV and the resulting pro-inflammatory state may contribute to dysregulation of finely tuned immune–bone interactions, ultimately leading to increased bone loss (McGinty et al., 2016). Hence, new therapeutics for osteoporosis are urgently needed.

Botanical drugs have been shown to exhibit various pharmacological activities in the treatment of osteoporosis. Corynoline, an isoquinoline alkaloid of *Corydalis bungeana*, possesses therapeutic potential in bone metabolism disease by inhibiting osteoclast formation and bone loss through modulation of the NF-κB/MAPK and Nrf2 signaling pathways (Jin et al., 2024). Icaritin, a major active metabolite of TCM formulations derived from *Epimedium*, offers various pharmacological benefits. Recent studies have reported that icaritin inhibits osteoclast differentiation by downregulating two transcription factors: nuclear factor of activated T-cell cytoplasm 1 (NFATc1) and c-fos (Huang et al., 2023). Ginseng-derived ginsenoside Rb2 contributes to osteoporosis by suppressing osteoclastogenesis and modulating NF-κB/MAPK signaling pathways in mice (Ma et al., 2024). Catalpol, a major active metabolite of *Rehmannia glutinosa*, reportedly attenuates bone loss by promoting osteoclast apoptosis via the Sirt6/ERα/FasL axis (Chen et al., 2024). In conclusion, TCM represents a promising therapeutic strategy for osteoporosis, although further studies are needed to elucidate the actions of herbal metabolites on bone remodeling.

### 2.9 Pathogenic mechanisms linking HIV to multisystem comorbidities

**FIGURE 1:**
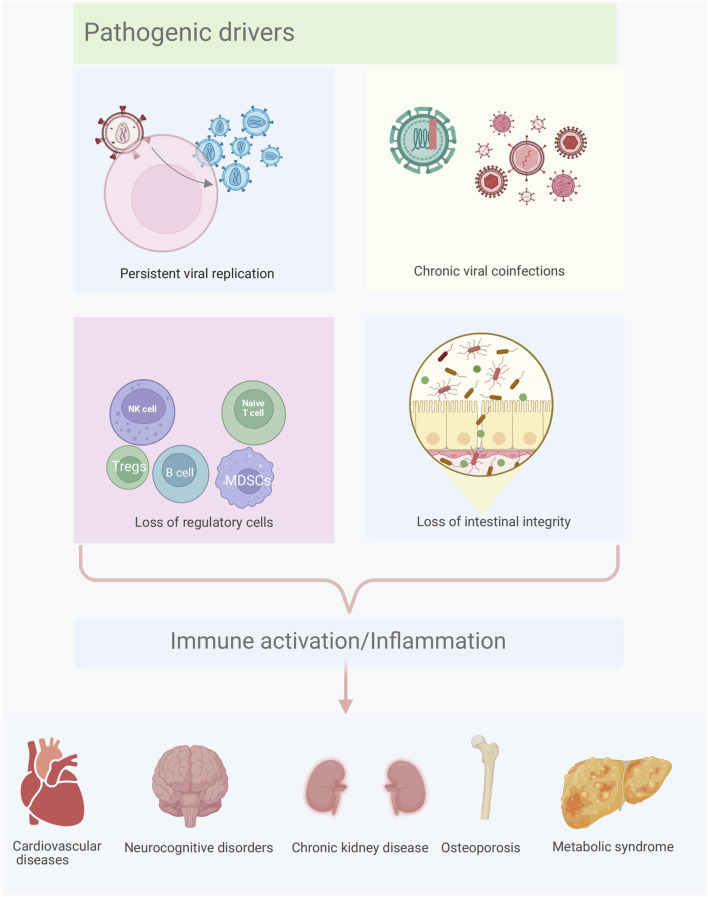
Pathogenic drivers and HIV-associated comorbidities. Pathogenic drivers include persistent viral replication, chronic viral co-infections, loss of regulatory cells, and loss of intestinal integrity. This persistent inflammatory and immune-activated environment increases their susceptibility to multiple comorbidities (cardiovascular diseases, neurocognitive disorders, chronic kidney disease, osteoporosis, and metabolic syndrome, among others).

Despite effective suppression of HIV replication with ART, persistent inflammation and immune activation remain common in adult PLWH, increasing their susceptibility to multiple comorbidities. The fundamental causes of immune activation/inflammation involve multiple pathogenic mechanisms, including persistent HIV production, co-infections, irreversible immune dysregulation, and intestinal microbial translocation (Deeks et al., 2013). This inflammatory environment leads to the development of comorbid illnesses, including CVDs, CKDs, MetS, osteoporosis, and neurocognitive disorders, through several potential pathways, ultimately damaging terminal organs (Figure 1).

### 2.10 Interference with HIV replication

Despite suppressive ART, HIV replication continues in PLWH because of the indefinite persistence of integrated viral genomes within CD4^+^ T cells and potentially other cell types (Cohn et al., 2020). Furthermore, the HIV-1 reservoir can be reactivated through pro-inflammatory cytokines and signals from damaged neurons, leading to intermittent cycles of viral expression and silencing in the brain (Sreeram et al., 2022). HIV-1 reservoirs are considered a major barrier to treatment. Strategies aimed at curing HIV-1 infection include interventions to eliminate the viral reservoirs or to enhance the immune responses that effectively control viral replication. Although the effects of botanical drugs on HIV-1 reservoirs have not been reported, some may have anti-HIV activities. Glycyrrhizin, the main active metabolite in Radix Glycyrrhizae, exerts antiviral activity. Intercellular adhesion molecules are crucial in HIV infection (de Gaetano Donati et al., 2004). Studies have shown that herpes simplex virus infection significantly increases the adhesion force and stress between cerebral capillaries in endothelial cells and polymorphonuclear leukocytes. Glycyrrhizin perfusion significantly inhibits this adhesion, suggesting an anti-inflammatory effect (Huang et al., 2012). Trichosanthin, extracted from the root of the Chinese medicinal plant *Trichosanthes kirilowii* Maximowicz, has been shown to induce apoptosis in JAR cells, enhance chemokine activity (regulated upon activation, normal T-cell expressed and secreted and stromal cell-derived factor (SDF)-1a-stimulated chemotaxis), and inhibit HIV-1 integrase (Shaw et al., 2005). Sparstolonin B, isolated from the Chinese medicinal plant *Sparganium stoloniferum*, was recently reported to block HIV-1 transcription via the transactivation response element region known as TAR (Deng et al., 2015). Further exploration of these therapeutic TCM approaches may be beneficial.

### 2.11 Inhibition of chronic viral co-infections

Common chronic viral co-infections caused by cytomegalovirus and HCV are associated with heightened T-cell activation, aggravating the inflammatory environment during ART (Hunt et al., 2011; Gonzalez et al., 2009). New treatment programs designed to control these concurrent viral infections are needed. Curcumin extracted from *Curcuma longa* exhibits anti-human cytomegalovirus activity by targeting heat shock protein 90 (Lv et al., 2015). The methanolic extract from Rhizoma Coptidis reportedly blocks HCV attachment and entry/fusion into host cells (Hung et al., 2018). Schisandronic acid from *Schisandra sphenanthera* inhibits the entry of pan-genotype HCV into human liver cells by interfering with viral particle–cell membrane fusion (Zhang et al., 2021). Celastrol extracted from *T*. *wilfordii* Hook F. inhibits HCV translation and the inflammatory response marker NLRP3 by specifically targeting heat shock protein 90β (Chen SR. et al., 2023). These metabolites may serve as candidate therapeutics for managing HIV-related chronic viral infections.

### 2.12 Regulation of regulatory T-cell (Treg) loss

Recent studies have shown that pathogenic viral infection is associated with rapid depletion of T helper 17 (Th17) cells and an increased frequency of Tregs (Kanwar et al., 2010). Th17/Treg imbalance is a hallmark of HIV infection and a marker of disease progression. This dysregulation contributes to immune dysfunction and microbial translocation, driving chronic immune activation, systemic inflammation, and disease progression (Wacleche et al., 2017). Natural plant extracts have gained considerable attention due to their natural origin, efficacy, and safety. *Toddalia asiatica* extract (alkaloids and coumarins) can inhibit the expression levels of Th17-related proteins and mRNAs (IL-17A, RORC, IL-1β, and IL-6) while increasing the expression levels of Treg-related proteins and mRNAs (IL-10 and FOXP3), which aids in restoring the balance of Th17/Treg (Qin et al., 2023). Aloperine, which is extracted from *Sophora alopecuroides* L., can modulate the Th17/Treg balance through promoting the conversion of Th17 to Treg and the generation of Tregs via altering the pSTAT3/pSTAT5 ratio (Zhou et al., 2022). The extract of *Lindera aggregata* (Sims) Kosterm. can significantly modulate the Th17/Treg balance by suppressing IL-6 differentiation and regulating the IL-6/STAT3 signaling pathway (Lai et al., 2021). In addition, some botanical drugs have been reported to reduce chronic inflammation and immune activation by regulating Th1/Th2 imbalance (Li et al., 2022; Ji et al., 2014; Zhao et al., 2019). These findings represent new evidence supporting the application of botanical drugs in the treatment of HIV.

### 2.13 Maintenance of the intestinal barrier

HIV destroys the CCR5^+^ CD4^+^ T cells present in gut-associated lymphatic tissue, leading to the loss of intestinal integrity. This allows intestinal microbiota products to be transported into the bloodstream, leading to systemic inflammation and immune activation (Mak et al., 2021; Santinelli et al., 2023). PLWH have been found to exhibit increased levels of lipopolysaccharide (LPS), an indicator of microbial translocation, along with systemic LPS-positive bacterial extracellular vesicles, both of which can induce immune activation (Tulkens et al., 2020). During HIV infection, the diversity of the intestinal microbiota decreases, along with a loss of beneficial bacteria and an increase in some potential pathogens (Lozupone et al., 2013). This microbial imbalance is associated with the impairment of intestinal barrier integrity. Additionally, HIV-related microbial dysbiosis is conducive to the production of pro-inflammatory cytokines, including IL-6, IL-17, and TNF-α, which activate Th1 and Th17 cells and intensify inflammation and tissue damage (Islam et al., 2024; Baldelli et al., 2021). Microbial translocation resulting from intestinal barrier destruction further amplifies systemic inflammation and immune activation (Farcomeni et al., 2021). Maintaining the integrity of the intestinal mucosa and the balance of intestinal flora is critical for preventing the pathogenic processes that drive HIV infection.

Botanical drugs have been widely used to maintain intestinal mucosal integrity in HIV infection. Isovitexin, isolated from *Thlaspi arvense*, aids in maintaining the intestinal mucosal barrier integrity by attenuating TNF-α-induced epithelial damage (Wang et al., 2025). Berberine, the active metabolite of *Coptis chinensis* and *Berberis* spp., protects the intestinal epithelial barrier from inflammatory response-induced injury through activation of the AKT1/SOCS1 pathway and reduced levels of the pro-inflammatory cytokines TNF-α and IL-10 (Liu et al., 2018; Hering et al., 2012; Li et al., 2010). Berberine also ameliorates intestinal injury by inhibiting the proliferation of Th1 and Th17 cells (Li et al., 2015). Polysaccharides derived from *Pyrus pashia* Buch.-Ham induce intestinal mucosal damage repair processes by inhibiting the expression of inflammatory genes through the MAPK/NF-κB pathway, attenuating the release of inflammatory cytokines (TNF-α, IL-6, and IL-1β) while enhancing the expression of tight junction proteins (ZO-1, occludin, and claudin-1). Furthermore, these polysaccharides were shown to restore the diversity of the intestinal microbiota and increase beneficial short-chain fatty acids (Zhang et al., 2025). Polyphyllin VI derived from *Rhizoma Paridis* decreases intestinal epithelial barrier damage via autophagic modulation of the NLRP3 inflammasome (Yuan et al., 2025). Thus, botanical drugs may represent a new strategy for maintaining intestinal barrier integrity.

Side effects of prolonged ART, such as toxicity and lipodystrophy, pose a potential threat to the long-term success of ART and the ultimate elimination of AIDS (Imahashi et al., 2021). Studies have reported that certain botanical drugs can alleviate ART-related toxicity and lipodystrophy; however, the underlying mechanisms require further exploration (Liya et al., 2015; Jian et al., 2020).

## 3 Conclusion

The high prevalence of co-infections underscores the need for enhanced prevention and management strategies for HIV-related comorbidities, and a multidisciplinary approach remains crucial for reducing the burden of complex, overlapping conditions in PLWH. A growing body of evidence supports the increasing use of botanical drugs by both practitioners and patients, who value their affordability, convenience, and beneficial therapeutic effects on AIDS-related comorbidities. Botanical drugs offer prevention and treatment of a range of AIDS-related chronic comorbidities, including CVDs, CKD, neurocognitive disorders, MetS, osteoporosis, and neurocognitive disorders, through their anti-inflammatory and anti-immune activation effects.

Preclinical evidence supports the effectiveness of some Chinese botanical drugs preparations as complementary and alternative therapeutic options; however, most of the available evidence is based on *in vitro* and animal studies, and their clinical translational value is significantly limited. Further rigorous clinical trials are needed to verify the efficacy and safety of Chinese botanical drugs in treating PLWH. The animal models are of limited value because they do not fully replicate the complexity of human HIV infection, the effects of ART, and polygenic settings. In addition, more rigorous evaluations of the included studies are required, especially regarding potential biases, specific dose/concentration details, and strict standardization of plant materials. However, data on the effects of botanical drugs in treating HIV-related chronic comorbidities are limited. First, the pharmacology and therapeutic mechanism of botanical drugs and their active metabolites have not been extensively explored, and further research to optimize their clinical application is required. Second, further research on the potential drug–drug interactions between metabolites of botanical drugs and ART is warranted. Lastly, future mechanistic studies with deeper insights into key molecular targets and dose–response relationships are anticipated. In conclusion, botanical drugs hold great promise as complementary therapeutic options in the management of HIV-related chronic comorbidities, which warrants further exploration.
